# Single-Cell Transcriptomics in Cancer Immunobiology: The Future of Precision Oncology

**DOI:** 10.3389/fimmu.2018.02582

**Published:** 2018-11-12

**Authors:** Fatima Valdes-Mora, Kristina Handler, Andrew M. K. Law, Robert Salomon, Samantha R. Oakes, Christopher J. Ormandy, David Gallego-Ortega

**Affiliations:** ^1^Genomics and Epigenetics Division, Garvan Institute of Medical Research, Darlinghurst, NSW, Australia; ^2^St. Vincent's Clinical School, Faculty of Medicine, University of New South Wales, Sydney, NSW, Australia; ^3^The Kinghorn Cancer Centre, Garvan Institute of Medical Research, Darlinghurst, NSW, Australia; ^4^Garvan-Weizmann Centre for Cellular Genomics, Garvan Institute of Medical Research, Darlinghurst, NSW, Australia

**Keywords:** ScRNA-seq, tumor immunology, MDSCs, tumor heterogeneity, stroma, single-cell transcriptomics

## Abstract

Cancer is a heterogeneous and complex disease. Tumors are formed by cancer cells and a myriad of non-cancerous cell types that together with the extracellular matrix form the tumor microenvironment. These cancer-associated cells and components contribute to shape the progression of cancer and are deeply involved in patient outcome. The immune system is an essential part of the tumor microenvironment, and induction of cancer immunotolerance is a necessary step involved in tumor formation and growth. Immune mechanisms are intimately associated with cancer progression, invasion, and metastasis; as well as to tumor dormancy and modulation of sensitivity to drug therapy. Transcriptome analyses have been extensively used to understand the heterogeneity of tumors, classifying tumors into molecular subtypes and establishing signatures that predict response to therapy and patient outcomes. However, the classification of the tumor cell diversity and specially the identification of rare populations has been limited in these transcriptomic analyses of bulk tumor cell populations. Massively-parallel single-cell RNAseq analysis has emerged as a powerful method to unravel heterogeneity and to study rare cell populations in cancer, through unsupervised sampling and modeling of transcriptional states in single cells. In this context, the study of the role of the immune system in cancer would benefit from single cell approaches, as it will enable the characterization and/or discovery of the cell types and pathways involved in cancer immunotolerance otherwise missed in bulk transcriptomic information. Thus, the analysis of gene expression patterns at single cell resolution holds the potential to provide key information to develop precise and personalized cancer treatment including immunotherapy. This review is focused on the latest single-cell RNAseq methodologies able to agnostically study thousands of tumor cells as well as targeted single-cell RNAseq to study rare populations within tumors. In particular, we will discuss methods to study the immune system in cancer. We will also discuss the current challenges to the study of cancer at the single cell level and the potential solutions to the current approaches.

## Introduction

In the last few years the use of single cell transcriptomics for the understanding of complex biological systems has boomed, producing remarkable insights in the fields of immunology, neurobiology, or cancer biology (1–3). Because of the outstanding prospective to reveal new cell types and states, the analysis of gene expression profiles at the single cell resolution has an exceptional potential to understand the complex interconnections that occur in the tumor microenvironment. In this review, we take a journey on the study of tumor immunobiology one cell at the time, discussing different approaches, technologies and providing a glimpse of the achievements that single-cell RNAseq will bring in the near future.

## Cancer heterogeneity and the immune system

### Tumor microenvironment

**Figure 1:**
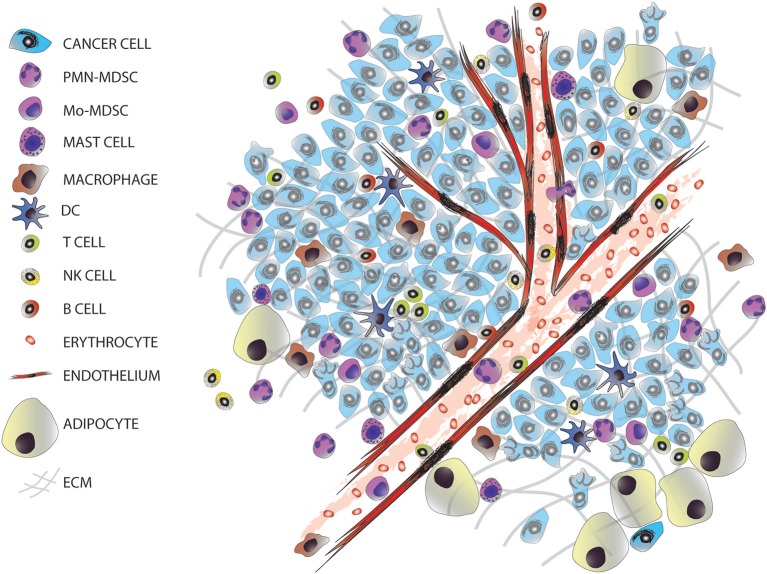
The tumor microenvironment. Tumors are entities formed by different cell types, including many infiltrated cells from the innate and adaptive immune system.

The majority of solid tumors have an epithelial origin but tumors are not exclusively formed by epithelial cancer cells, they consist of a complex and heterogeneous conglomerate of multiple cell types from different origins, which can be divided into two groups: cancer cells transformed from the epithelium of the tissue of origin; and stromal cells, comprised of seemingly normal tissues including fibroblasts, adipocytes, endothelial, and immune cells (Figure 1). This later group, together with the extracellular matrix, forms the tumor microenvironment (TME). TME is comprised of highly diverse cell types that have clearly contributed to the hallmarks of cancer (4, 5). The stromal cells can be classified into: infiltrating immune cells (IICs), endothelial vascular cells, and cancer-associated fibroblasts (CAFs). These stromal cells within the TME contribute to seven out of the eight acquired hallmarks of cancer: (1) the evasion of growth suppressors; (2) sustaining proliferative signaling; (3) the resistance to cell death; (4) reprogramming energy cellular metabolism; (5) the initiation of angiogenesis; (6) the evasion of the immune destruction; and (7) the induction of invasion and metastasis (4).

### The role of the immune system in cancer

#### Major mechanisms of action of the immune system in cancer

**Figure 2:**
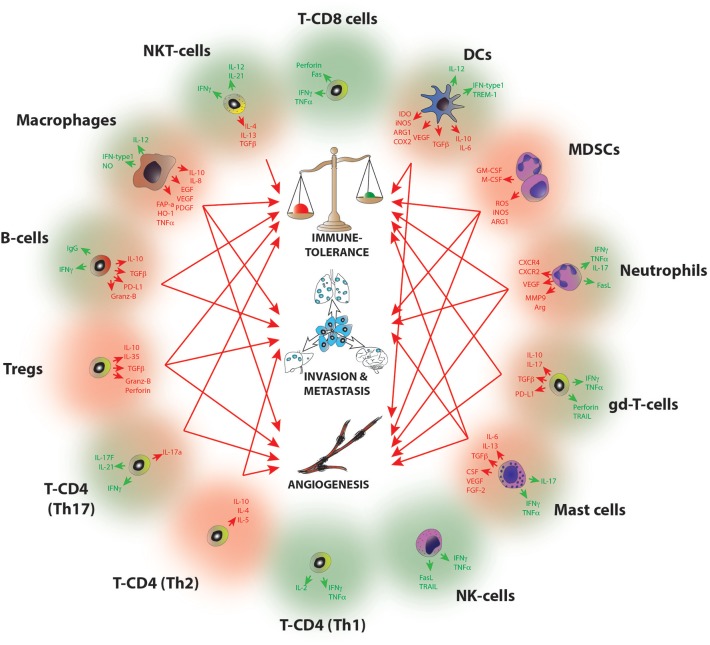
Molecular pathways of tumor progression driven by IICs. Diagram summarizing the main pro-tumorigenic (red) and anti-tumorigenic (green) mechanisms exerted by infiltrated immune cell species. Red arrows indicate the hallmarks of cancer progression where each cell has been implicated, B-cells (I, M); Mast cells (I,A,M); Macrophages (I,A,M); Dendritic cells (I,A); MDSCs (I, A, M); Neutrophils (I, A,M); NKT (I); gd-T cells (I, A); Th2 T-CD4 (A,M); Th17 T-CD4 (I,A); Tregs (I,A,M). I, Immune tolerance; A, Angiogenesis; M, Metastasis. For a full reference please see Table 1.

A common feature of all cancers, regardless of its origin, is the presence of different clusters of immune cells (6) (Figure 1). Due to the intense heterogeneity of the IICs these cells are able to influence on the fate of cancer cells in many different and contrasting ways (Figure 2 and Table 1). For example, in general many tumor types with extensive infiltration of pro-inflammatory immune cells and CD8^+^ T lymphocytes have better prognosis and, in contrast, tumors with high presence of immunosuppressive IICs like regulatory T lymphocytes or myeloid-derived suppressor cells show worse outcomes [reviewed in Barnes et al. (54)]. Research has focused on trying to unravel how the different subtypes of IICs are selected or recruited to tumors to explain this dichotomy [reviewed Palucka and Coussens (55)]. These studies have come to the conclusion that it is the specific intercommunication among cancer cells, the TME and immune cells that determines the balance toward immunotolerance or immunerejection. In fact, these interactions are a dynamic “yin-yang” process, where the immune system is able to recognize cancer cells as “foreign” to suppress the progression of cancer; while a sustained anti-tumor immune response will trigger mechanisms designed to prevent tissue damage and to maintain tissue homeostasis by inhibiting natural killer (NK) cells and effector T cells. This ultimately produces the suppression of the immune system that subsequently allows the cancer to escape the immune control, bypass apoptotic pathways and maintain inflammation and angiogenesis (56).

**Table 1:** Pro-tumourigenic and anti-tumourigenic functions of immune cells^*^.

**Immune cell**	**Tumor promotion**	**Tumor suppression**	**Cancer type**	**References**
B cells	•Secretes cytokines (IL-10, TGF-β, lymphotoxin), immunosuppressive molecules (PD-L1, granzyme B) •Inhibit T-cell activation and CD4^+^ T cell response •Promote metastasis and angiogenesis •Suppress CD8^+^ T cell and NK cell anti-tumor activity •Recruit myeloid cells antibodies to induce immunosuppressive environment	•Promotes CD4^+^ and CD8^+^ activity •Prolongs T cell survival •Secrete cytokine (IFNγ) and IgG •Recruit NK cells to eradicate cancer cells •Enhance antibody-dependent cell-mediated cytotoxicity (ADCC) by NK cells and phagocytosis in macrophages •Act as antigen presenting cells to stimulate anti-tumor response	•Bladder •Breast •Cervical •Colorectal •Diffuse large B-cell lymphoma •Gastric •Glioblastoma •Head and neck •Immunoblastic sarcoma •Leukemia •Liver •Lung •Melanoma •Myeloma •Non-Hodgkin lymphoma •NSCLC •Ovarian •Pancreatic •Prostate •Renal cell carcinoma •Squamous cell carcinoma •Thyroid	(7–9)
CD4^+^ Th1 cells		•Promotes CTLs survival and activity •Recruit and activate antigen-presenting cells, NK cells, and M1 •cell infiltration •Secretes cytokines (IFNγ, TNF-α, IL-2) to Inhibit angiogenesis and induce cancer cell apoptosis	•Bladder •Breast •Cervical •Cholangiocarcinoma •Colorectal •Gastric •Glioblastoma •Head and neck •Leukemia •Liver •Lung •Melanoma •Myeloma •Non-Hodgkin lymphoma •NSCLC •Ovarian •Pancreatic •Prostate •Renal cell carcinoma •Squamous cell carcinoma	(10–14)
CD4^+^ Th17 cells	•Secrete cytokine (IL-17A) •Suppresses immune response and promotes angiogenesis •Recruits immunosuppressive	•Activates CTLs •Secretes cytokines (IFNγ, IL-17F, IL-21)	•Bladder •Breast •Cervical •Colorectal •Gastric •Glioblastoma •Head and neck •Leukemia •Lung •Melanoma •Non-Hodgkin lymphoma •NSCLC •Ovarian •Pancreatic •Prostate •Renal cell carcinoma	(10, 13–15)
CD4^+^ Th2 cells	•Cytokine production (IL-10, IL-4, IL-5) •Inhibit CTL proliferation	•Recruits M1 for arginase-mediated cancer eradication in adoptive cell therapy •Priming of CTLs	•Bladder •Breast •Cervical •Cholangiocarcinoma •Colorectal •Gastric •Glioblastoma •Head and neck •Leukemia •Liver •Lung •Melanoma •Myeloma •Non-Hodgkin lymphoma •NSCLC •Ovarian •Pancreatic •Prostate •Renal cell carcinoma •Squamous cell carcinoma	(10, 11, 13, 14, 16)
CD4^+^ Treg cells	•Exhaustion of T-cell and NK activities •Inhibit expansion of CD4^+^ and CD8^+^ T cells •Suppresses antigen-presenting cell activities and expression of antigen-presenting molecules and inflammatory cytokines •Inhibit B cell antibody production •Strongly promotes immunosuppressive tumor environment •Induces ATP catabolism •Secretes cytokines (IL-10, TGF-β, IL-35), immunosuppressive molecules (granzyme B, perforins)	•Regulate inflammation to restore immune response	•Bladder •Breast •Cervical •Cholangiocarcinoma •Colorectal •Gastric •Glioblastoma •Head and neck •Hepatocellular carcinoma •Leukemia •Lung •Melanoma •Mesothelioma •Myeloma •Non-Hodgkin lymphoma •NSCLC •Ovarian •Pancreatic •Papillary thyroid •Prostate •Renal cell carcinoma •Squamous cell carcinoma	(11, 17–19)
CD8^+^ cells	•Activity inhibited by immunosuppressive environment and cells	•Perforin or Fas-mediated cytotoxic response to cancer cells •Secretes cytokines (IFNγ, TNF-α)	•B-cell non-Hodgkin lymphoma •Bladder •Breast •Cervical •Cholangiocarcinoma •Colorectal •Gastric •Glioblastoma •Head and neck •Hodgkin Lymphoma •Leukemia •Liver •Lung •Lymphoma •Melanoma •Myeloma •NSCLC •Ovarian •Pancreatic •Prostate •Renal cell carcinoma •Squamous cell carcinoma	(12, 20, 21)
Dendritic cells	•Secretes cytokines (TGF-β, IL-10, IL-6), growth factors (VEGF), immunosuppressive molecules/enzyme (arginase, iNOS, IDO, COX2) •Reduced priming of CD8^+^ T cells in response to elevated levels of B-catenin •Direct suppression of CD8^+^ T cell activity by arginase and Stimulate Treg formation	•Assists in priming of CD4^+^ and CD8^+^ T cells through tumor antigens •Adaptive immune response •Secretes cytokines (IL-2, IL-12, type I IFN), T-cells costimulatory molecules and cytokines for Th1 and Th17 priming (TREM-1) •Links the innate and adaptive immunity as antigen-presenting cells	•Bladder •Blastic plasmacytoid dendritic cell neoplasm •Breast •Cervical •Colorectal •Gastric •Glioblastoma •Head and neck •Hepatocellular carcinoma •Lung •Melanoma •NSCLC •Ovarian •Pancreatic •Prostate •Renal cell carcinoma •Squamous cell carcinoma •Thyroid	(11, 22–26)
Macrophages	•Pro-tumourigenic TAM (M2) •Secretes cytokines (TGF-β, IL-10, IL-8), growth factors (VEGF, EGF, PDGF), immunosuppressive molecules (FAP-a, HO-1), and ECM remodeling (MMP-9) •Direct immunosuppression of CD8+ T cells •Induces EMT •Enhance tumor cell migration and pre-metastatic niche •Promote angiogenesis and metastasis	•Anti-tumourigenic (M1) •Secretes cytokines (IL-12, type I IFN) and NO •Eliminate cancer cells through phagocytosis and cytotoxicity	•Bladder •Bone metastasis •Breast •Cervical •Cholangiocarcinoma •Colorectal •Gastric •Glioblastoma •Head and neck •Leukemia •Liver •Lung •Melanoma •NSCLC •Ovarian •Pancreatic •Prostate •Renal cell carcinoma •Sarcoma •Squamous cell carcinoma •Thyroid	(10, 11, 27–31)
Mast cells	•Secretes cytokine (IL-6, IL-13, CSF), growth factors (TGFβ, VEGF, FGF-2), protease (tryptase) •Promote metastasis and Enhance immunosuppressive effects of MDSC and inflammation from other immune cells	•Recruit and activate T cells and DC •Secrete cytokines (IFNγ, TNF-α)	•Bladder •Breast •Cervical •Cholangiocarcinoma •Colorectal •Diffuse large B-cell lymphoma •Gastric •Glioblastoma •Head and neck •Hepatocellular carcinoma •Leukemia •Lung •Melanoma •NSCLC •Ovarian •Pancreatic •Prostate •Renal cell carcinoma •Sarcoma •Squamous cell carcinoma •Thyroid	(32–34)
MDSC	•Exhaustion of T-cell activities •Induce NK anergy •Inhibit T-cell infiltration •Induces TAM differentiation •Strongly promotes immunosuppressive tumor environment •Induces EMT •Enhance tumor cell migration and preparing the pre-metastatic niche •Secrete (M-CSF), growth factors (VEGF), immunosuppressive molecules (ROS, iNOS, ARG1)		•Bladder •Breast •Cervical •Colorectal •Gastric •Glioblastoma •Head and neck •Hepatocellular carcinoma •Hodgkin's lymphoma •Leukemia •Lung •Melanoma •Non-Hodgkin's lymphoma •NSCLC •Ovarian •Pancreatic •Prostate •Renal cell carcinoma •Sarcoma •Squamous cell carcinoma •Thyroid	(10, 11, 35)
Neutrophils	•Pro-tumourigenic TAN (N2) in late tumourigensis •Expression of cytokines (TGFβ, CXCL8) growth factors (VEGF), (arginase, ROS), ECM remodeling (MMP-9) •Promotes angiogenesis, metastasis, cancer cell proliferation •Induce NK anergy	•Anti-tumourigenic (N1) during early tumourigenesis •Secrete cytokines (IFNγ, TNFα) •TRAIL and FasL-mediated apoptosis of cancer cells •apoptosis of cancer cells •Antibody-dependent cell-mediated cytotoxicity (ADCC) of cancer cells •Elimination of tumor cells during early tumourigenesis activate T Cells	•Bladder •Breast •Cervical •Cholangiocarcinoma •Chronic neutrophilic leukemia •neutrophilic leukemia •Colorectal •Gastric •Glioblastoma •Head and neck •Liver •Lung •Melanoma •Non-Hodgkin lymphoma •NSCLC •Ovarian •Pancreatic •Prostate •Renal cell carcinoma •Squamous cell carcinoma •Thyroid	(36–40)
NK cells	•Rapid death of NK cells through lack of IL-2 and IL-15	•ADCC, FasL, TRAIL-mediated apoptosis of cancer cells •Secretes cytokines (IFNγ, TNFα) •Promote DC maturation and elimination of immature DC •Cross-talk between macrophages, NKT, DC, and T-cells for synergise anti-tumor response	•Anaplastic thyroid cancer •Bladder •Breast •Cervical •Cholangiocarcinoma •Colorectal •Gastric •Glioblastoma •Head and neck •Liver •Lung •Melanoma •Natural killer cell leukemia •Natural •NSCLC •Ovarian •Pancreatic •Prostate •Renal cell carcinoma •Squamous cell carcinoma •carcinoma	(41–44)
NKT cells	•Secretes cytokines (IL-4, IL-13, TGFβ) •Suppress Type I NKT and CD8^+^ T cell anti-tumor activity	•Type I NKT cells produces cytotoxic response to cancer cells •Recruits and activates Th1 cells •Abolish immunosuppressive functions in TAM, TAN and MDSC •Secretes cytokines (IFNγ, IL-12, IL-21)	•Bladder •Breast •Cervical •Cholangiocarcinoma •Colorectal •Gastric •Glioblastoma •Head and neck •Liver •Lung •Melanoma •Myeloma •Natural killer/T-cell lymphomas •NSCLC •Ovarian •Pancreatic •Prostate •Renal cell carcinoma •Squamous cell carcinoma	(45–50)
γδ T cells	•Secrete cytokines (IL-10, IL-17, TGFβ) and immunosuppressive molecules (PD-L1) •Inhibit DC activities and T-cell differentiation •Induce immunosenescence in DC and T-cells •Promote immunosuppression and angiogenesis •Recruitment of MDSC and	•Perforin, TNF, TRAIL mediated cytotoxic response to cancer cells •Secretes cytokines (IFNγ, TNF-α) •Links the •Links the innate and adaptive immunity as antigen-presenting cells •Enhance antibody production through interaction with B-cells •Promote DC maturation and differentiation of naive CD4^+^ T cells to CTL •Improve NK cell cytotoxicity against cancer cells	•Bladder •Breast •Cervical •Colorectal •Gastric •Glioblastoma •Head and neck •Leukemia •Lung •Lymphoma •Melanoma •Myeloma •NSCLC •Ovarian •Pancreatic •Prostate •Renal cell carcinoma •Squamous cell carcinoma	(51–53)

* *Immune cell can have both pro-tumourigenic and anti-tumourigenic activity based on tumor type, microenvironment, stage, localization, and immune subset*.

Multiple pathways of immunesuppression take place in the TME (Table 1), and they involve different immune cell subtypes including but not restricted to Th2-polarized macrophages, myeloid-derived suppressor cells (MDSCs) and regulatory T cells (Tregs) (Figures 1, 2). TME and cancer cells are responsible for producing chemokines and signaling pathways to block the effector function of T cells (e.g., checkpoints that control cytotoxic T-cell differentiation and function) and to attract immunosuppressive immune cells to the tumor. For example, intratumoural C-C motif chemokine 22 (CCL22) is induced in tumor-infiltrating dendritic cells through interleukin-1 alpha (IL-1α) secreted by cancer cells; the accumulation of CCL22 induces the recruitment of regulatory T cells through the expression of CC chemokine receptor 4 (CCR4) and these tumor-infiltrating Tregs suppress local effector T cell responses (57).

As mentioned above, IICs are indirectly involved in seven out of eight acquired cancer hallmark capabilities (55), but also they drive two major “direct” pro-oncogenic mechanisms that lead to tumor progression: chronic inflammation and immune tolerance.

##### Chronic inflammation network

The onset of inflammation and its duration is a critical feature in the balance between immune rejection and immune tolerance. In fact, inflammation is a hallmark of cancer where immune cells have either pro- or anti-tumor properties (5). In this context, there are contrasting findings regarding the impact of the immune system in inflammation, for example, it has been shown that chronic inflammation is a risk factor for cancer development (58) but on the other hand treatment with bacterial mixtures that induce acute inflammation led to tumor regression in sarcomas (59). These paradoxical properties of leukocytes are due in part to their functional plasticity and this makes them an attractive cancer therapeutic target (6). Cancer-associated inflammation encompasses a broad spectrum of immune cells (both myeloid and lymphoid) and soluble factors (Figure 2), such interacting networks will be discussed below in a cell-subtype basis.

##### The immunosuppressive tumor network

Tumors have developed mechanisms to create an immunosuppressive environment enriched for soluble mediators, receptors and cells. In fact, TME contains high leukocytic infiltrations that present immunosuppressive activities similar to the wound-healing stage: they are able to block CTLs, in the case of cancer anti-tumor CTLs; or inhibit Natural Killer T (NKT) cells that in the neoplastic context mediate the elimination of cancer cells. These immunosuppresive IICs comprise Tregs, MDSCs, tumor-associated macrophages (TAMs, programmed by Th2-type cytokines), tolerogenic dendritic cells (DCs), regulatory B cells and mast cells (60). Thus, despite the intense presence of tumor-infiltrating T cells (TIL) in many cancers, often their cytotoxic activity is impaired by the presence of these immunosuppressive IICs and in turn these TILs are not able to successfully attack cancer cells.

#### Immune cell subtypes with pro-tumoral characteristics

Here, we present a summary of the most important myeloid and lymphoid populations responsible of an immune response to cancer and how their physiological processes are exploited by cancer cells to escape immunosurveillance (Figure 2 and Table 1). Consequently, these IICs have become clear candidates for an effective immunotherapy in cancer.

##### Myeloid compartment (innate response)

Macrophage progenitors can differentiate to become alternatively activated TAMs when exposed to Th2 cytokines (e.g., IL-4, IL-13) and other factors (e.g., thymic stromal lymphopoietin, immune complexes) (10). TAMs are one of the main components of the leukocytic infiltrate in tumors (61), these are usually M2 like macrophages with the capability to promote tumourigenesis. Some of the mechanisms include: (1) the production of growth and survival factors of tumor cells [transforming growth factor beta (TGFβ), epidermal growth factor (EGF), IL-6, etc.]; (2) production of angiogenic factors [vascular endothelial growth factor (VEGF), platelet-derived growth factor (PDGF), IL-8, etc.]; (3) degradation of the extracellular matrix; (4) activation of tissue remodeling; and (5) suppression of adaptive response by the production of immunosuppressive factors [IL-10, prostaglandin E2 (PGE2), etc.], the reduction of immunostimulatory cytokines (IL-12) and releasing chemokines (CCL17, CCL18, etc.) to recruit Tregs (27, 28).

MDSCs represent a heterogeneous population composed of precursors of the myeloid-cell lineage that resemble immature neutrophils. Pro-inflammatory cytokines skew myeloid cell differentiation and perturb their maturation, resulting in a spectrum of immature myeloid cells (IMC) that are morphologically analogous to granulocytes and monocytes (62, 63) but can be distinguished from these cells by their potent immunosuppressive activities (64). MDSC are recruited to the tumor site by growth factors and pro-inflammatory molecules secreted by cancer cells; for example tumor-produced granulocyte-macrophage colony stimulating factor (GM-CSF) recruits and accumulates MDSC in the TME through GM-CSF receptor (65); tumor-produced VEGF is also able to chemoattract MDSC through the expression of VEGF receptor (66); S100A4 and A9 are soluble factors produced by the cancer cells that attract MDSCs through the receptor for advanced glycation end products (RAGE) (67) and, more recently, Blattner and colleagues have shown that melanoma cells are able to recruit C-C chemokine receptor type 5 (CCR5)-positive MDSCs into the TME through the production of its ligands (IL-6, GM-CSF, CCL3/4/5) (68, 69). Once recruited and expanded in the TME, MDSCs are able to induce NK cell and T-cell anergy, angiogenesis, and epithelial-mesenchymal transition (EMT). As immature myeloid precursors, MDSCs are very plastic and can be programmed *on site* to specific phenotypes and functions by the signals present in the TME (70). MDSCs were originally described as cells that potently suppress both innate and adaptive anti-tumor immunity. MDSCs inhibit T cells (both CD8^+^ and CD4^+^) by producing arginase I (ARG I) and reactive oxygen species (ROS) and through the induction of nitric oxide synthase expression (71); but also suppress NK and NKT cells and inhibit DCs maturation (71–73). It is now clear that the contribution of MDSCs to tumourigenesis is not restricted to immune-suppression and includes regulation of tumor growth, progression, the formation of the pre-metastatic niche, and metastasis (74, 75). Tumor activated MDSC infiltrate in normal organs and assist in establishing a premetastatic niche, supporting seeding of metastatic cells by promoting their survival and suppressing immune rejection (76–80). The specific tumor-derived soluble factors that induce MDSC-migration, aberrant activation and expansion are still largely unknown. Clinically, increased circulating MDSC correlated with poor patient prognosis and survival (81–83).

Tumor-infiltrated DCs are defective functional mature DCs that are unable to properly stimulate the immune system as a result of the significantly increased myelopoiesis that takes place in cancer (84). In addition, many soluble factors present in the TME affect DC differentiation and function including VEGF, macrophage colony-stimulating factor (M-CSF), IL-6 and accumulation of adenosine and hypoxia (85). Hypoxia-inducible factor-1 (HIF-1) activates DCs to up-regulate the adenosine receptor, which activates Th2 cells (86, 87). Adenosine-activated DCs express pro-inflammatory IL-6, pro-angiogenic VEGF, and immunosuppressive mediators IL-10, cyclooxygenase 2 (COX2), TGFβ and indoleamine 2,3-dioxygenase (IDO) (22).

##### Lymphoid compartment (adaptive response)

The mechanisms used by the cells involved in an adaptive response are summarized in Table 1 and Figure 2. In this section we reviewed the most common pro-tumourigenic lymphocytes subtypes found in cancer.

Despite the critical role of T lymphocytes in immune surveillance and control of early tumor growth, later sustained tumor cell and TME secretion of cytokines and other soluble factors with pro-tumourigenic/immunosuppresive capabilities, alter T cell function and recruitment (88–90).

Tregs cells are CD4^+^ T lymphocytes characterized by the expression of the FoxP3 transcription factor that can also be identified by the expression of CD25 and CD127 in humans. Tumor-derived factors can promote the recruitment and expansion of Tregs. This T cell subtype is able to suppress excessive immune responses to pathogens, a mechanism that is widely adopted by cancer cells (17, 18). Tregs are able to polarize immunity away from an anti-tumor response, block CD8^+^ T cell activation and NK cell “killing” activity (19). High Treg–CD8 ratios in tumor infiltrates correlate with poor patient survival (91).

NKT cells are a subclass of T cells that express natural killer cell surface markers. Type II NKT cells have been reported to down-regulate tumor immune surveillance and suppress anti-tumor responses. Type II NKT cells are activated by endogenous ligands, such as lysophosphatidylcholines (92), which initiate the production of IL-4, IL-13, and TGF-β. The presence of these factors block CTL and NK cell functions (45, 46, 93). IL-13 secretion, via the IL-4R–STAT6 signaling pathway, can induce production of the pro-tumourogenic and pro-metastatic TGF-β-producing MDSCs (47).

### The use of immunotherapy in cancer

The immune system is a key player in both pro-tumor and anti-tumor responses (94). Due to its plasticity, cancer cells have developed mechanisms to skew immune responses toward tumor immune tolerance to create an immunosuppressive environment. Thus, the re-education of the immune system toward tumor rejection is a very promising strategy for cancer therapy (95). An immunotherapy strategy would ideally require a combined stimulation of potent anti-tumor immune response and the eradication or reprogramming of the immunosuppressive environment. In fact, a few immunotherapy approaches have produced very impressive results in some cancer types like lung cancer and melanoma (96). Despite these exciting results, there are still unresponsive patients and tumor types where immunotherapy is not effective (97). Therefore, understanding the role of IICs in different tumor types and patients is currently a field of intense research. Different immunotherapy-based strategies are the focus of the current research, this includes: (1) neutralization of chronic tumor inflammation (6); (2) cancer vaccination to increase T-cell repertoires, both DC-based vaccination (98) and adoptive T-cell transfer (99), which could be dramatically enhanced when combined with strategies to eliminate or differentiate MDSCs (63, 100); (3) To identify inducers of immunogenic cell death (ICD) to re-activate immune rejection through the production of damage-associated molecular patterns (DAMPs) from the stressed or dying cancer cells (101); and, (4) elucidating the mechanisms and cell identity of the IICs that regulate tumor tolerance and cytotoxic T-cell activity (MDSC, T-regs, NKT cells).

Direct and indirect immunotherapeutic strategies that target immunosuppressive cells are now in the clinic. For example, Ipilimumab or Nivolumab are monoclonal antibodies that are being used to target the checkpoint pathway of CTLs [antagonize cytotoxic T-lymphocyte-associated protein 4 (CTLA-4) and Programmed Death 1 (PD-1), respectively] (102). The inhibition B7 family members and/or their ligands (PDL-1 and PDL-2) have a huge potential use in the clinic as they are expressed in many immunosuppressive IICs like Treg cells, MDSCs, tolerogenic DCs, and TILs (103).

Another mechanism of immune activation with a potential use in cancer therapy is ICD. Radiotherapy and some chemotherapeutic agents [e.g., Doxorubicin (104) or Vorinostat (105)] induce ICD in cancer cells. These dying cancer cells release factors and/or express danger molecules so called DAMPs as a consequence of the activation of ROS and endoplasmic reticulum stress pathways. These DAMPs act as adjuvants being able to stimulate anti-tumor immune responses and subsequently to elicit tumor rejection (101). Thus, the identification of specific ICD inducers and the activation pathways within the cancer cells to actively produce DAMPs is also a very active area of research for immune-based cancer therapy.

Altogether a combinatorial therapy to target both cancer cells and cells from the TME or their mediators (including immunotherapy) will constitute a synergistic strategy toward a potent cancer treatment response. Therefore, a complete understanding of the mechanisms and immune cell diversity and their molecular activation status in each patient and tumor type are crucial in order to design personalized and targeted immune-based therapy.

## Heterogeneity of IICs

It is evident that a myriad of complex interactions exists between cancer cells and immune cells that are pivotal for both pro-tumourigenic and anti-tumourigenic roles (56, 106, 107) (Figure 2 and Table 1). As such, tumor infiltrated immune cells are highly heterogenous (Figure 1), and this complexity is amplified by the dynamic interactions that can occur among cancer cells, immune cells and the TME. This high heterogeneity explains in part the development of resistance to immunotherapy as the current targets might not fully trigger all the immunesuppresive IICs or potentiate the anti-tumor effect of the right effector T cells.

Bulk transcriptome profiling of IICs has been critical to identify plausible novel targets, to understand their pro/anti-oncogenic function and their association with patient survival (108–110). For instance, both TILs and TAMs are relatively abundant populations in many cancers and as such are amenable for study using conventional transcriptomic analysis techniques. This has probably contributed to the high number of studies addressing the role of these populations in cancer (108–110). For example, a study by De Simone and colleagues analyzed the transcriptome of isolated TILs (Tregs, Th1 and Th17 T cells) from lung and colorectal tumors. They found that TILs are molecularly different from peripheral T cells, suggesting an active re-education by the TME and cancer cells. They also found that a Treg-gene signature correlated with poorer patient outcome in both cancer types (108). Despite the advantages offered by bulk transcriptome analyses, this methodology is not sufficient to uncover the full spectrum of immune diversity within tumors (2) and in turn this has made the development of novel immunotherapies very challenging (97). In addition, the study of underrepresented and/or highly heterogeneous tumor infiltrated immune populations has challenged the conventional bulk transcriptome analyses. Recent research on TILs using single-cell RNAseq (scRNAseq) has shown that this methodology offers superior understanding of cell diversity (111–117). The finding of these studies are further discussed below and clearly demonstrates that scRNAseq is an exceptionally suited technology for the understanding of the contribution of IICs different populations to tumor initiation and progression (2).

### Heterogeneity of low abundant populations: the MDSCs example

MDSCs are a low abundant and highly heterogeneous tumor infiltrated immune population with strong relevance in the cancer progression. In mice, CD11b and Gr-1 surface markers define the MDSC immunosuppressive cell population (118, 119). Gr-1 expression can be then subclassified using the Ly6G and Ly6C epitopes. Thus, CD11b^+^Ly6G^+^Ly6C^low^ MDSCs correspond to polymorphonuclear granulocytes (PMN-MDSC), while CD11b^+^Ly6G^low^Ly6C^+^ MDSCs present monocytic morphology (M-MDSC). Various reports indicate that these two populations might have distinct functions in infectious and autoimmune diseases, graft vs. host disease, and cancer (75, 120); where they display various functions depending on the tumor type, disease stage, factors present in the TME and anatomical location. Despite considerable efforts and progress using marker-based approaches, most of MDSC heterogeneity is not explained by these markers and is not truly compatible with any of the myeloid lineage classification schemes. Thus, the main characteristic to define the identity of MDSCs in cancer is their immunosuppressive capacity (71), however recent studies have demonstrated a ROS-mediated anti-metastatic role of tumor-associated neutrophils in mice bearing 4T1 mammary tumors (121). This new data suggests a possible divergent polarization or differentiation of neutrophils and that organ and tumor-specific myeloid cells have major differences in their molecular phenotypes and CTL suppressive activity. Altogether, these contrasting findings show that the identity of the MDSC subsets in tumors and their molecular mechanism of interaction with the TME, including their pro or anti-tumourigenic role, remain to be discovered. Due to their low abundance and their extensive heterogeneity, single-cell molecular profiling of tumor infiltrated MDSCs is an excellent suited methodology for the understanding of this population.

## The use of transcriptomics to study cancer

Transcriptomic analysis of bulk tumor populations is a very powerful tool to study cancer. In fact, the analysis of 44 drug sensitive predictive algorithm by the National Cancer Institute and the Dialogue on Reverse Engineering Assessment and Methods (NCI-DREAM) project has demonstrated that gene expression profiling is superior on predicting drug sensitivity and its power increases when including multiple independent datasets (122).

The introduction of international cancer consortiums like The Cancer Genome Atlas (TCGA), that contain publicly available information on clinical, pathological transcriptomic, genomic, and methylation data of multiple human tumor types, has resulted in a stellar acceleration of findings (123, 124). This database has enabled the stratification of samples into tumor subtypes; the prediction of patient prognosis, drug-resistance, and response to treatment; and a deeper understanding of tumor biology (125–127). These studies have also shown that “omic” heterogeneity exists within individual tumor types and a comprehensive understanding of such heterogeneity is crucial for cancer treatment (128). Thus, the response rate to targeted therapies increase when a stratification based on the molecular characteristics of the tumors is applied, for example the use of imatinib in chronic myelogenous leukemia (129) or estrogen antagonists for estrogen-receptor-positive breast cancers (130).

### Molecular portraits of breast cancer as a lead example

Breast cancer is a highly heterogenous disease; as such its subclassification has been under intense research in the last two decades. In the clinic, breast cancer is classified according to the presence of three nuclear receptors: human epidermal growth factor receptor 2 (HER2), estrogen receptor (ER) and progesterone receptor (PgR). The presence of the biomarkers in immunohistochemical analyses classifies breast cancer into 3 different subtypes: ER+, Triple Negative (TNBC), and HER2+ breast cancers. This subclassification determines therapeutic intervention, for example anti-estrogen therapy for ER+ tumors or chemotherapy for the TNBC. However, within each group there are still differential responses to treatment, for example about 1/3 of the ER+ patients will become resistant to anti-estrogen therapy. In 2000, Perou and colleagues published a seminal paper describing a new molecular classification of breast cancer subtypes based on significant differences in their gene expression profiles (131). This study was the beginning of a tsunami of papers on gene expression analyses in hundreds of breast cancer samples as a tool for differential diagnosis, prognosis and prediction of therapeutic sensitivity (132–137). In 2009, Parker et al. created a minimal gene set called PAM50 to stratify “intrinsic” subtypes of breast cancer (138). This PAM50 signature is now been commonly used to subdivide breast cancer patients as it has shown a strong agreement with other larger “intrinsic” gene sets previously used for subtyping. Functional modules using transcriptional profiles are also informative of patient prognosis (139), thus, the most informative parameters in the ER+ subtype is related to proliferation, further classifying this subtype into luminal A and B, the latter highly proliferative and associated to poorer prognosis. In contrast, in the TNBC subgroup, only the immune response module was associated with prognosis, whereas in the HER2+ tumors, the tumor invasion and immune response modules displayed significant association with survival.

With the advance of next-generation technologies and the formation of large consortiums like TCGA, now it is possible to have a comprehensive genome-wide transcriptomic (RNAseq), genomic (exome or DNA sequencing), and epigenomics (Methyl-seq) data in hundreds of tumor samples (140, 141). The integration of this information is an invaluable source to explain breast cancer heterogeneity. For example, exome sequencing, Methyl-seq, gene expression (mRNA and miRNA), and protein expression on more than 800 breast cancer patients identified more than 30,000 genomic mutations (141) and interestingly these authors found that of all the breast cancer subtypes, the Basal-like subtype has more molecular similarities to high-grade serous ovarian cancer than any other breast cancer subtypes. These new results suggest that Basal-like breast cancer patient could benefit from drugs commonly use to treat ovarian cancer (e.g., PARP inhibitors) and illustrates the potential of the implementation of genomic analysis for the clinical management of cancer.

### Single-cell RNA-sequencing in cancer

These advances in next generation sequencing methods have been key in the new era of precision medicine that is rewriting clinical cancer treatment. However, there is still significant percentage of patients that do not respond to molecularly designed personalized therapies even when their tumors are cataloged based on both molecular and pathologic criteria. One plausible explanation to this matter is that all of these analyses are based on bulk tumor data. This *omic* information of ensembles of cells is dominated by the most abundant cell populations, normally the cancer cells, and masks the *omic* profiles of low abundant or rare populations, including rare cancer cell types with differentiated properties such as cancer initiating cells, but also cells from the TME. Differential transcriptional programs explain much of the functional cell diversity and also provide information on the possible interactions of the cancer cells with the TME. In fact, the transcriptomic information of the cells from the TME will provide invaluable information to design new targets on these cells to use in combination with conventional drugs that target the cancer epithelial cells. Thus, single cell characterization of cancer will allow a more precise characterization and stratification of patients according to single cell “*omic”* information, and holds the potential for the development of novel and more targeted molecular therapies. The characterization of IICs could also identify better strategies to target immunosuppressive signaling or ICD pathways aimed to potentiate natural cancer immunerejection and immune surveillance.

In the last few years, advances in single cell isolation and the reduction of sequencing costs has allowed an stellar growth in methods to analyse the genome, transcriptome, and epigenome at the single cell level (142). Of the vast array of single cell genomic technologies currently available, single-cell RNAseq (scRNAseq) is currently the most informative and robust method to understand the biology of lowly represented cell populations within the tumor. Single-cell RNAseq can also illustrate tumor paracrine-signaling networks, and can be used as a strategy to develop combined therapies to target multiple and relevant cell populations within tumors.

Initially, scRNAseq allowed the analysis of dozens of cells, which due to subsampling limitations and the high heterogeneity of tumors generated a very poor understanding of TME biology and rare populations. The introduction of targeted scRNAseq (143), droplet-based and other high-throughput scRNAseq methods (144–148) and the reduction of the sequencing costs (149) has enabled the analysis of thousands of tumor cells. Thus, in the last 5 years, studies on single-cell transcriptome of tumors of different cancer types have emerged, this includes (Table 2): glioma (150, 151, 157), melanoma (112), colorectal cancer (159), hepatocellular carcinoma (113, 152), renal carcinoma (153), non-small-cell lung cancer (NSCLC) (115, 116, 154), breast cancer (111, 114, 117, 148, 156), and myeloid leukemia (155).

**Table 2:** Summary of the studies in human tumors using scRNAseq.

**Cancer type**	**Tumor cell types**	**scRNAseq method**	**Cell number**	**References**
Glioblastoma	Cancer epithelial cells	Smart-seq	430	(150)
Oligodendrogliomas	Cancer epithelial cells	Smart-seq2	4,347	(151)
Hepatocellular cancer	Cancer epithelial cells	scTrio-seq	25	(152)
Renal carcinoma	PDX	Fluidigm C1/SMARTer	116	(153)
Lung adenocarcinoma	PDX	Smart-seq	34	(154)
Chronic myeloid leukemia	Lin^−^CD34^+^CD38^−^ cells	*BCR-ABL*-targeted Smart-seq2	2,000	(155)
Breast cancer (TNBC)	Cancer epithelial cells	Nanogrid single-nucleus RNA seq	7,278	(148, 156)
Hepatocellular cancer	CD8^+^ and CD4^+^ T cells	Smart-seq2	5,063	(113)
Gliomas	Inter (CD11b^+^) and Intra tumor TAMs (*in silico*)	10X Genomics	4,039	(157)
Gliomas	Inter (CD11b^+^) and Intra tumor TAMs (*in silico*)	Fluidigm C1/SMARTer	466	(157)
Non-small-cell lung cancer	CD3^+^ TILs	Smart-seq2	12,346	(115)
Breast cancer (TNBC)	CD3^+^ TILs	10X Genomics Chromium	6,311	(117)
Breast cancer (ER^+^PR^+^, Her2^+^, TNBC)	Immune cells (CD45^+^)	inDrop	47,016	(114)
Breast cancer (ER^+^PR^+^, Her2^+^, TNBC)	CD3^+^ TILs	10X Genomics Chromium (scRNA-seq and paired V(D)J sequencing)	27,000	(114)
Melanoma	All cell types in tumor	Smart-seq2	4,645	(112)
Head and neck cancer	All cell types in tumor	Smart-seq2	5,902	(158)
Breast cancer(ER^+^PR^+^, Her2^+^, TNBC)	All cell types in tumor	Fluidigm C1/SMARTer	515	(111)
Colorectal cancer	All cell types in tumor	Fluidigm C1/SMARTer	590	(159)
Non-small-cell lung cancer	All cell types in tumor	10X Genomics Chromium	92,948	(116)

#### Single-cell transcriptomics of the epithelial compartment from human tumors

Initial scRNAseq studies on human tumors were exclusively focused on the malignant epithelial compartment with the aim to study intratumoural heterogeneity of cancer cells. The major motivation of these studies was to identify subpopulations of epithelial cells or rare populations of tumor initiating cells that could explain resistance to targeted therapy, overcoming the limitations of bulk RNA-seq or microarray studies. The very first study of scRNAseq in tumor samples was done by Patel et al. (150). A total of 430 single cells were isolated from five primary glioblastomas and full-length mRNAseq was performed using the Smart-seq approach (160). Their major findings showed an extremely high intratumoural heterogeneity at different levels: (1) Mosaic expression for typical receptors and ligands from glioblastoma-related pathways that have been used as targets for therapy, e.g., EGFR or receptor tyrosine kinases. (2) Gradient expression of stemness and differentiation cell states, and (3) Expression of markers of different glioblastoma subtypes within the same tumor sample. All of these heterogeneous features have a direct impact on prognosis prediction and therapeutic strategies. This study uncovered the heterogeneity of tumor cells of human glioblastoma, however these tumor samples were depleted of tumor-infiltrated leukocytes (CD45^+^) prior scRNAseq and then the downstream analyses were only focused on cancer cells [420 cells with cancer-related copy number variations (CNVs)] thus a bigger picture of tumor heterogeneity beyond cancer cells could not be established. Despite of this limitation, the authors identified TME-related signaling pathways in the cancer cells, including immune response and hypoxia pathways (150). Two years later, Tirosh et al were able to increase the number of single cells analyzed by 10 times (4,347 cells from six human oligodendrogliomas) using Smart-seq2 protocol (149, 151, 161). The authors identified for the first time a rare subpopulation of undifferentiated cells associated with a neural stem cell expression program. These cells were classified as cancer initiating cells that represent promising targets to affect tumor growth for this incurable glioma subtype. Although this study initially did not deplete the tumor infiltrated leukocyte CD45^+^ population, non-malignant cells were subsequently excluded by estimating CNVs, so their downstream bioinformatic analyses were only focused on the cancer cells and the authors did not perform further analyses on the cells from the TME (303 cells in total).

In another study in hepatocellular cancer, 25 single cells were analyzed by RNAseq using the Tang-Surani method (152, 162). Despite of the low number of cells analyzed in this study the real advantage was the development of a novel method to simultaneously analyse DNA copy number, DNA methylome, and transcriptome (scTrio-seq) in single cells that allows a more comprehensive analysis of tumor heterogeneity. Thus, this multiomic analysis revealed two subpopulations with differences in CNVs, DNA methylation, and RNA expression profiles, in which the lowest represented subpopulation showed more malignant markers, this include more gain of CNVs, more gene signatures of cell invasion and evasion of immune surveillance. Again however, this study exclusively focused on the cancer cells and excluded cells from the TME.

Kim et al. analyzed a total of 116 cells from a single metastatic renal carcinoma patient using C1™ Single-Cell Auto Prep System (Fluidigm) followed by SMARTer kit [commercial kit based on Smart-seq (160)] (153). This study focused on the differences between primary and metastatic tumor sites in order to identify intratumoural heterogeneity to explain drug resistance. Single-cell analyses showed the presence of subpopulations with distinct drug sensitivities between the primary and metastatic sites, an also among individual cancer cells in each tumor location. They went one step further and showed that the combinatorial drug treatment targeting those differential pathways significantly improved therapeutic responses in both *in vitro* and *in vivo* models. This study was also limited by the lack of information on the TME for several reasons, the first one is that the only looked at around 30–40 cells per tumor site/model which it will give an extremely low representation of the cells from the TME (which normally accounts 30–40% of the total cells from the tumor); and the second reason comes from the origin of the tumor sample, where 82 cells came from patient-derived xenografts (PDXs): 36 cells from PDXs from the metastatic site and 46 cells from PDXs from the primary tumor (153). PDXs are useful models to have “unlimited” tumor samples from a given patient and useful for *in vivo* drug screening (163, 164). PDX cells reflect their parental tumors but with lower normal stromal cells content, which is replaced by mouse stroma and gets diluted out throughout the xenograft passages. The second limitation is that human cancer cells grow in immunocompromised hosts, thus this model cannot be used to study the effect of the immune system on tumor progression or cancer therapy (153). The same authors did a very similar study but this time using exclusively PDXs from a lung adenocarcinoma patient tumor where they identified a candidate tumor cell subgroup associated with anti-cancer drug resistance (154), hence this study presents the same limitation on studying the TME involvement in drug resistance.

Another study analyzed more than 2,000 single cells by FACS and Smart-seq2 from patients with chronic myeloid leukemia (CML) at diagnosis, remission and disease progression (155). In addition, this team developed a new method to simultaneously obtain high-sensitivity mutation detection of *BCR-ABL* (a fusion gene present in CML and used as therapeutic target) and transcriptome analysis of the same single cell. This method was *BCR-ABL*-targeted Smart-seq2 protocol where *BCR-ABL*-specific primers were multiplexed at the reverse transcription and amplification steps. This technique revealed heterogeneity of CML single cells, including the identification of a subgroup of CML cells with a differential molecular signature that selectively persevered during sustained therapy.

Recently, the introduction of a novel scRNAseq methodology called *Nanogrid single-nucleus RNA sequencing* has allowed the analysis of isolated single nuclei from archived fresh frozen tumor tissues (148). The creators of this new method were able to sequenced the polyA mRNA from 416 nuclei in one study (148) and 6,862 nuclei in another one from frozen breast tumor samples (156). This latter study combined single cell DNA seq and scRNAseq to study the acquisition of chemoresistance in TNBC and found that while the genotype of resistant cells was pre-existent to neoadjunvant chemotherapy treatment, the transcriptome presented a very different transcriptional profile pre- and post- treatment (156). This study underscores the high plasticity of transcriptome in the development of chemoresistance that might correspond to the pre-existing resistant cancer cells. As such, these cells might be cancer-initiating cells present in the tumor since its inception and constitute the perfect targets to tackle chemoresistance in TNBC. One of the limitations of these studies is that the image system only selects the nuclei from cancer epithelial cell and excludes stromal cells (> 8 microns) and also the bioinformatics analyses only focused on aneuploidy cells to make sure none of the cell from the TME were included in their downstream analyses. Thus, any contribution of the TME to chemoresistance remains elusive.

#### Single-cell transcriptomics of IICs from human tumors

Recently large-scale studies focus on the tumor stroma and particularly in IICs have flourished. Zheng et al., Savas et al., and Guo et al. exclusively focused their studies on tumor-infiltrated T cells from liver cancer, TNBC, and non-small-cell lung cancer, respectively (Table 2). The common motivation underlying these studies was the identification new immunotherapeutic strategies for poorly responsive tumors to the current immunotherapy-based drugs, as well as for a better patient stratification to be selected for the current checkpoint inhibitor immunotherapy (113, 115, 117). Thus, all these three studies primarily found interesting results in CD8^+^ T cells and their exhaustion (lost of self-renewal capacity) and effector properties and in Tregs heterogeneity and their immunosuppressive activity.

In the liver cancer study, the transcriptome of 5,063 FACS isolated T cells (CD4^+^ or CD8^+^) from peripheral blood, tumor, and adjacent normal tissues from 6 hepatocellular carcinoma patients was sequenced (Smart-seq2). Transcriptional profiles of these individual cells, combined with single cell TCR sequences, identified 11 T cell subsets (5 clusters for CD8^+^ and 6 clusters for CD4^+^ cells) with diverse tissue distribution patterns, molecular characteristics and functional properties. Specific subsets of Tregs and exhausted CD8^+^ T cells were more abundant in the liver primary tumors compared to the T cells from the peripheral blood or adjacent normal tissue. They were also able to validate one novel gene signature in CD8^+^ T cells governed by the layilin gene (LAYN). The overexpression of LAYN in CD8^+^ T cells from normal peripheral blood resulted in inhibition of IFN-γ production (a cytokine involved in immune-mediated tumor rejection), this suggests that LAYN might be a master regulator of a major signaling pathway involved in the inhibition of the cytotoxic activity of CD8^+^ T cells and that it could mediate immunesuppression in the TME.

Savas et al. identified a very similar T cell heterogeneity (10 clusters) from 2 samples of human primary TNBC tumors where 6,311 tumor-infiltrated T cells were sequenced (117) using the 10X Genomic Chromium platform. The major findings of this study were the identification of a new subclass of tissue-resident memory CD8^+^ T cells (CD8^+^CD103^+^, T_RM_) that may be responsible of cancer immunesurveillance as these cells showed cytotoxic and pro-inflammatory characteristics both in the scRNAseq data as well as in *in vitro* functional assays. Furthermore, the gene signature of this T_RM_ cells was significantly associated with better patient prognosis. Altogether this study identified a T cell subpopulation resident in breast tumors as a potential new target of immune-intervention.

Guo and colleagues performed scRNAseq (Smart-seq2) in 14 naïve-treated NSCLC (115) identifying a higher T cell diversity than the previous two studies (7 CD8^+^ clusters and 9 CD4^+^ clusters). This study found two interesting CD8^+^ T cell clusters with pre-exhaustion markers that were associated with a significant better patient survival in lung cancer patients. A highly migratory T cell cluster that might be linked to a positive response to checkpoint inhibitors was also identified and lastly they also observed a very high heterogeneity within the Tregs subpopulations that might suggest different stages of activation.

There is one study that exclusively focuses on the other highly abundant IIC population in tumors, TAMs, in the context of gliomas, which are particularly abundant in this tumor type. Inter and intra-tumor TAMs were analyzed by two scRNAseq methods, C1 Fluidigm/SMARTer and 10X Genomics Chromium, (Table 2) and were also combined with public scRNAseq data from this tumor type (157). Interesting the authors found that blood-derived TAMs present a different gene signature than the microglial TAMs and that contribute to a more M2 phenotype (express immunesupressive cytokines and have an altered metabolism) for pre-treatment gliomas and its presence correlates with poorer survival in low-grade gliomas suggesting a potential use as a blood-based prognostic biomarker and suggest a more TAM-targeted immunotherapeutic strategy.

Recently Azizi et al. built the first human breast tumor immune single cell atlas sequencing the transcritpome of 47,016 immune cells from breast cancer tumors of different subtype origin (114) (Table 2). This very high resolution was only possible by the use of the inDrop (indexing droplet) platform (145) after FACS CD45^+^ cells from tumors. Their data revealed a very high heterogeneity of these cells where they were able to identify 83 clusters that correspond to 38 T cell (15 CD8^+^ and 21 CD4^+^), 27 myeloid cell, 9 B cell and 9 NK cell clusters. In-depth bionformatic analyses of the T cell and myeloid lineages, which were the most abundant and are the most clinically relevant, showed a higher diversity of these cell populations in the tumors when compared with their matched normal mammary tissue, peripheral blood or lymph nodes. Very interestingly they found that the TAMs present in the tumors have co-exiting gene signatures of M1 and M2 states suggesting that tumor-infiltrated macrophages are quite plastic and can exist along a continuum between 2 states, this has been also observed in gliomas (157) and NSCLC (116). Their comprehensive analysis on the T cells compartment also suggest a spectrum of activation transitions rather than classical discrete states and such stages are TCR-induced and tumor microenvironment-dependent.

All these studies in IICs underscore the capability of high-throughput scRNAseq methods on revealing the high complexity and diversity of the IICs in tumors; they are also an invaluable resource for future further mechanistic studies and for the development of novel immunotherapy strategies. In the future the combined information of the corresponding gene-signatures coming from the other stromal cells as well as from the cancer epithelial cells will also add further insight of how these different T cells and TAM transitions are regulated; this could be achieved using the same type of high-throughput scRNAseq platforms in a unbiased manner both in the single cell isolation and bioinformatics analysis steps. Another missing piece in these studies is a deeper analysis and/or focus on rare IICs that are clinically relevant like MDSCs, which with the current technology is now possible to obtain high-resolution transcriptome profiling of such low-abundant populations.

With the exponentially growing studies generating sc transcriptome data on IICs in different human cancer types it would be very interesting to combine this information to look for common immunogenic transcriptional signatures or immune cell subtypes involved in tumor immunesuppression or immunesurveillance for the design of globally effective immunotherapeutic treatments independent to tumor type and also to use this data for stratification of patient that could benefit of such treatments.

#### Unbiased single-cell transcriptomics of human tumors

In 2016, the studies of scRNAs-seq analyzing both malignant and stromal cells emerged. This has demonstrated that the inclusion the single cell transcriptome of cells from the TME added an important layer of information key for deeper understanding of the cancer ecosystem, for the design of novel TME-based therapeutic targets and to explain drug resistance, particularly, resistance to immunotherapy. Tirosh et al. analyzed 4,645 single cells from 19 melanoma patients with a variety of clinical and therapeutic backgrounds, profiling malignant cells (CD45^−^ and inferring CNVs) and non-malignant cells (CD45^+^ and inferring of CNVs): immune, stromal, and endothelial cells (112). These tumors were dissociated to single cells followed by FACS (alive and CD45 positive cells) and Smart-seq2 for single-cell RNAseq analyses. Globally, single-cell analyses of all cells suggested different TME profiles that highlighted cell-to-cell interactions between cancer and stromal cells with direct implications for both targeted and immune therapies. In particular, the authors found that malignant cells show high diversity in the abundance of a dormant and drug-resistant melanoma subpopulation that directly impacts on drug resistance. In the non-malignant subpopulation they identified six different cell subpopulations: T cells, B cells, macrophages, NK cells, endothelial cells, and CAFs. Single-cell expression analysis of 2,068 infiltrating T cells from 15 melanomas cells further allowed the definition of a core exhaustion signature for CD8^+^ T cells of 28 genes that were consistently increased in high-exhaustion T cells. Full-length scRNAseq is highly informative of TCR mRNA clonality in T cells, allowing the correlation of T cell clonal expansion with their exhaustion, in this study low exhausted T cells were practically non-expanded. These novel findings could be highly informative to evaluate tumor response and resistance to the current checkpoint inhibitors available in the clinic. This is the first study where the single-cell analysis of the cells from the TME are considered and showed for the first time the huge potential of scRNAseq in TME-cell types, in particular in the study of the differential transcriptional programs of the tumor-infiltrated T cells.

A study using similar approaches in head a neck squamous cell carcinoma (HNSCC) was published at the end of 2017 (158) where 5,902 cells from 18 HNSCC primary tumors and matching lymph node metastases were sequenced in an unbiased manner (FACS of alive cells). One of the highlights of this work was the discovery of complex interactions between malignant and non-malignant cells, in particular they found a paracrine crosstalk of partial EMT (defined as a signature with some EMT markers and moderated epithelial markers) between a subset of malignant cells from the leading edge of the tumor and CAFs from the TME with potential implications on tumor invasion. This work is particularly interesting as they are able to build interconnexions between the TME and epithelial cells and also to identify specific pathways of intra-tumor heterogeneity from the malignant cells regardless inter-tumor heterogeneity.

Another report contemplating all cell types in the tumor was published in the context of breast cancer (111). A total of 515 single cells from 11 patients representing the four subtypes of breast cancer (HER2+, luminal A, luminal B, and TNBC) were isolated using C1™ Single-Cell Auto Prep System followed by Smart-seq (SMARTer kit). This microfluidic-based method allowed marker-free isolation of malignant and non-malignant cells from tumors, obtaining 317 epithelial breast cancer cells, 175 tumor-associated immune cells and 23 non-carcinoma stromal cells. The analysis of breast cancer cells revealed a high grade of intratumour heterogeneity with the HER2 and TNBC subtypes showing the highest heterogeneity on their transcriptional programs. Interestingly, they identified rare populations of cells that expressed markers of aggressiveness, however not major conclusions from these cells could be drawn due to the low number of cancer cells analyzed per patient (<25). The investigation of the infiltrated immune cells revealed that the TNBC had the highest number of IICs compared to the other subtypes and that the mostly represented IIC subpopulations were: B cells (often from samples containing lymph nodes), T cells and TAMs (both mostly found in the primary tumors). Interestingly, the transcriptional programs of the T cells from the TNBC showed signatures of T-cell exhaustion but with low expression level of PD-1, suggesting the need to target different checkpoint molecules. In the same breast cancer subtype TAM subpopulations expressing many immunosuppressive M2-type genes were found. This study acknowledges the necessity of large-scale single-cell gene expression profiling projects for the comprehensive characterization malignant and non-malignant cells from heterogeneous tumors.

Li and colleagues performed scRNA–seq analysis (based on Smart-seq) of 590 cells (after quality control) isolated with the C1™ System from 11 primary colorectal cancer (CRC) tumors (375 cells) and matched normal samples (215 cells) (159). Besides the analysis of the cells from the TME, this study has the advantage of comparing matched normal samples that allow a more accurate differential expression analyses between normal and cancer cells coming from the same patient. scRNAseq data was able to stratify the tumors into cancer epithelial cells, fibroblasts, endothelial cells and major immune cell populations, some of which could be further divided into novel subtypes. In fact, two distinct subtypes of CAFs were identified where epithelial–mesenchymal transition-related genes were found to be up-regulated only in one CAF subpopulation. Thus, these results further underscore the potential relevance of this important cell type from the TME to CRC outcome.

This year, a seminal paper on high-resolution single cells profiling of NSCLC tumors has set the first stepping-stone toward the generation of reference atlas of high-resolution unbiased maps of tumors (116). Lambrechts and colleagues sequenced the single-cell transcriptome of NSCLC and matching adjacent normal tissue at the highest resolution ever reached before in tumors (Table 2). A total of 92,948 single cells (52,698 cell in a first cohort and 40,250 cells in a validation cohort) were subjected to unbiased scRNAseq (10X Genomics Chromium) and identified a total of 52 stromal cell subtypes of 12 cancer cell subtypes. Their analyses mostly focused on the TME due to several reasons: (1) Stromal cells showed higher than expected heterogeneity and complexity, even in cell types that are suppose to be homogeneous like endothelial cells; (2) These cells clustered according to cell type and, in contrast to the cancer epithelial cells, did not show any patient-bias; (3) TME is the most unexplored area of research in the context of tumors and sc transcritomics. As an example of one of their major findings they found that tumor endothelial cells showed up-regulation of Myc target genes that correlated with a higher global transcription of these cells and suggesting a role for Myc in angiogenesis. These tumor endothelial cells also present down-regulation of immune activation and immune cell homing suggesting an active role of this cell type in tumor immunetolerance. This study also briefly associated the tumor characteristics (histology and stage) with the TME using single-cell RNAseq information, correlating particular TME signatures or cell types/states with lung cancer subtypes [lung adenocarcinoma (LUAD) and lung squamous carcinoma (LUSC)]. In this context they identified 9 out of 42 TME cell subtypes that showed differences between lung cancer subtypes, that could be explained by the cell of origin of lung tumors, histopathology and stage. They also found that one of the CD8^+^ T cell clusters with high proliferative characteristics were positively associated with mutational load, suggesting a more T CD8^+^ effector response due to a higher number of tumor neo-antigens; the rest of the stromal clusters/signatures were correlated with a reduced mutational rate. However, more comprehensive functional associations between TME-cancer epithelial cells using scRNAseq information from both of them could have been challenging due to the high patient-to-patient bias in the epithelium compartment.

In conclusion, during 2018 we are witnessing a tsunami of new studies in scRNAseq of human tumors reaching a very high resolution and contemplating all cell types in the tumor (Table 2) that will lead to the establishment of single cell atlases of human tumors. These catalogs will set up the reference for further advances in cancer cell biology after functional validation *in vitro* and *in vivo* and will fuel major progresses in cancer diagnosis and therapy.

The application of high-throughput scRNAseq in pre-clinical models is also going to revolutionize the areas of cancer cell biology, cancer diagnosis and therapy. These models will be particularly useful for the study of TME-cancer epithelial interactions as mouse model are genetically homogeneous and controlled and allow further functional validation studies and *in vivo* drug testing.

## ScRNAseq methods to study IICs

Initially the most common scRNAseq methods to study human tumors are all based on Smart-seq (160) or its latest update Smart-seq2 (149) after single cell isolation by C1™ Fluidigm system or targeted FACS isolation, which limited the cell number throughput (Tables 2, 3). These methods allow targeting and sequencing of full-length transcripts using Illumina-based sequencing (149). Cells are sorted into 96 well plates containing lysis buffer and oligo-d(T) primers for reverse transcription. cDNA is amplified via polymerase chain reaction (PCR) and tagmentation (fragmentation and adapter ligation) is used for library preparation. All the molecular pipeline is performed cell by cell individually and samples are only pooled after adapter ligation for Illumina sequencing, thus the cost per cell is high and the throughput limited. These methods have allowed the analysis of up to ~5,000 single-cells from tumors [with the exception of one study where 12,000 cells were performed (115)], however due to the high heterogeneity of tumors, an increase of cell numbers from each compartment is crucial in order to have a enough resolution to resolve cell states as well as of low represented cell populations (cancer initiating cells or MDSCs). The introduction of cell barcoding prior to library preparation or even prior retro-transcription has significantly increased the throughput of scRNAseq enabling the simultaneous analysis of thousands of cells in a cost-effective manner (Tables 2, 3). Thus, studies analyzing tens of thousands of cells in human tumors (114, 116) have just emerged that are now more focused on the heterogeneity of cells from the TME. Thus, in the coming years there is an expectation of an explosion of new studies on high-throughput scRNAseq in whole tumors that will allow the generation of high-resolution human tumor atlases and the analysis of rare cell populations within tumors.

**Table 3:** Comparison of scRNAseq methods for the analysis of IICs.

**Assay**	**Cell capture strategy**	**cDNA amplification**	**Gene coverage**	**Throughput**	**Comparative cost**	**Advantage(s)**	**Limitation(s)**	**Reference(s)**
Drop-Seq	Droplet-based microfluidics	PCR amplified	3′ end of RNAs Poly(A)+ RNAs only	10^4^-10^5^	Low	High-throughput	3′bias No detection of poly(A)- RNAs and transcript variants Not suitable for FACS sorted cells	(144)
InDrop	Droplet-based microfluidics	IVT	3′ end of RNAs Poly(A)+ RNAs only	10^3^-10^5^	Low	High-throughput; Suitable for FACS sorted cells	3′bias No detection of poly(A)- RNAs and transcript variants	(145)
Chromium from 10X Genomics	Droplet-based microfluidics	PCR amplified	3′ end of RNAs Poly(A)+ RNAs only	10^4^-10^5^	High	High-throughput Easy handling	3′bias No detection of poly(A)- RNAs and transcript variants.	(165, 166)
MARS-Seq	Plate based	IVT	3′ end of RNAs Poly(A)+ RNAs only	10^2^-10^3^	Medium	Suitable for FACS sorted cells	3′bias Medium-throughput No detection of poly(A)- RNAs and transcript variants	(143)
CEL-Seq2	Plate based	IVT	3′ end of RNAs Poly(A)+ RNAs only	10^2^-10^3^	High	Suitable for FACS sorted cells	3′bias Medium-throughput No detection of poly(A)- RNAs and transcript variants	(167)
Smart-seq2	Plate based	PCR amplified	Full length RNAs Poly(A)+ RNAs only	10^2^-10^3^	Very high	Full length RNA and high number of genes per cell detected Detection of transcript variants Suitable for FACS sorted cells	Low-throughput No detection of poly(A)- RNAs	(149)
Nanogrid single-nucleus RNAseq	High-density plate	PCR amplified	3′ end of RNAs Poly(A)+ RNAs only	10^4^	Low	Image monitored and active doublet exclusion mechanism.	3′bias No detection of poly(A)- RNAs and transcript variants Requires specific equipment	(148)
Seq-well	Nanowell arrays sealed with a semipermeable membrane	PCR amplified	3′ end of RNAs; poly(A)+ RNAs only	10^4^-10^5^	Low	High-throughput Semipermeable membrane allows rapid solution-exchange, while trapping biological macromolecules Suitable for FACS sorted cells	3′bias Requires specific equipment No detection of poly(A)- RNAs and transcript variants	(147)
Microwell-Seq	Agarose-constructed microwell array	PCR amplified	3′ end of RNAs; poly(A)+ RNAs only	10^5^	Low	High-throughput; Image monitored and active doublet exclusion mechanism;	3′bias Requires specific equipment No detection of poly(A)- RNAs and transcript variants	(146)
DroNc-Seq	Droplet-based microfluidics	PCR amplified	3′ end of RNAs; poly(A)+ RNAs only	10^4^-10^5^	Low	Suitable for frozen tissues and tissues that cannot be dissociated Suitable for FACS sorted cells; Suitable for multi-omics (transcriptomics and proteomics)	3′bias Requires nuclei isolation prior to single cell capture; No detection of poly(A)- RNAs and transcript variants	(168)
Sci-RNA-Seq	Plate based (methanol-fixed cells or extracted nuclei)	PCR amplified	3′ end of mRNAs Poly(A)+ RNAs only	10^4^-10^5^	Low	High-throughput Fixed and frozen cells Suitable for FACS sorted cells; Suitable for multi-omics (transcriptomics and proteomics)	3′bias No detection of poly(A)- RNAs and transcript variants	(169)
SPLiT-Seq	Plate based (pooled formaldehyde-fixed cells or nuclei)	PCR amplified	3′ end of mRNAs Poly(A)+ RNAs only	10^4^-10^5^	Low	High-throughput Fixed and frozen cells Suitable for FACS sorted cells; Suitable for multi-omics (transcriptomics and proteomics)	3′bias No detection of poly(A)- RNAs and transcript variants	(170)

Droplet-based scRNAseq methods allow high-throughput analyses ideal to obtain high-resolution molecular phenotyping of complex tissues. These high-throughput technologies meet the need of extensive sampling of cells necessary to understand the complexity present in the TME; allowing the study of the signals arising from low-abundant populations. Thus, this technology is especially well-suited to unravel the contribution of tumor infiltrated immune cell populations involved in the onset of cancer.

**Figure 3:**
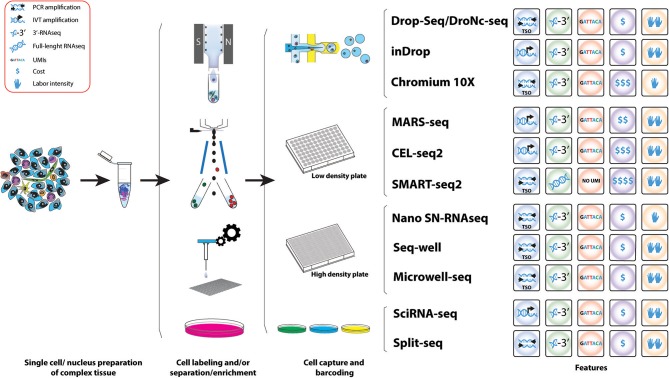
Workflow of various methods used for the study of IICs. Schematic representation of the different methods for scRNAseq analysis useful for the study tumor infiltrated immune cell species. A comparative summary of their main characteristics is shown.

Because of their potential, droplet-based scRNAseq have experimented a rapid technological development in the last years, resulting in the establishment of a number of different platforms. These methods present advantages and limitations and the choice of platform needs to be tailored to the scientific question aimed to answer; sample characteristics and abundance of the cell type of study. Here we provide an overview of the scRNAseq methods most suitable for analyzing the role of the immune system in cancer (Figure 3) including their advantages and limitations (Table 3). For example, the use of agnostic scRNAseq approaches without isolation bias maximizes the chance for novel cell type identification in tumor-associated subpopulations that may reveal new target populations for therapeutic intervention. This contrasts with, antibody-based isolation methods prior to scRNAseq, which are limited by preconceived cell marker information and the availability of high-affinity antibodies.

The major challenge of scRNAseq compared to bulk RNA-Seq is the minute amount of starting material, a mammalian cell only contains 1–50 pg of RNA and only 1–5 per cent are transcribed to mRNA (171). This problem has been overcome from two different angles: (1) by whole-transcriptome amplification of cDNA by PCR or RNA by *in-vitro* transcription (IVT), and (2) the introduction of random oligonucleotide barcodes that enable processing and sequencing of pools of barcoded cells in single reactions, but yet allowing assigning identified genes to their cell of origin. The disadvantage of these molecular methods is that individual cells need to be contained, normally in micro-wells, thus limiting the number of cells to be analyzed at same time as these approaches are highly time-consuming.

### Droplet-based microfluidic ScRNAseq methods

The development of droplet-based microfluidic single-cell RNA-Seq approaches like Drop-Seq and inDrop, has allowed high throughput capture and barcoding of tens-of-thousands of single cells in a short time, followed by pooling the extracted barcoded material on a single molecular reaction, massively reducing the cost per cell. In both techniques polyadenylated (poly(A)) RNAs are captured and barcoded with a cell barcode and a unique molecular identifier (UMI) (144, 145). These are unique oligonucleotide sequences that can hybridize to RNA via a poly(T) anchor. Transcripts of a single cell receive the same cell barcode but UMIs are unique throughout transcripts, which allows to account for PCR amplification biases (172).

In Drop-Seq, single cells are encapsulated into nanoliter-sized oil droplets containing lysis buffer and oligo barcodes attached to Toyopearl HW-65S beads. At encapsulation, single cells are lysed and RNA is barcoded by binding to the Poly(T) tails of the oligos on the beads producing so called STAMPs (single-cell transcriptomics attached to micro particles). The droplet emulsion is then broken and beads in suspension are recovered allowing a single reverse transcription reaction. cDNA is then amplified via PCR using a TSO primer (161). Amplified PCR products undergo tagmentation (fragmentation and adapter ligation) and library amplification before sequencing and bioinformatics analysis (144).

InDrop is based on the same principle of capturing single cells in droplets containing barcodes and UMIs, but there are some differences. Individual cells are encapsulated with hydrogel (polyacrylamide) microspheres containing barcoded primers in a solution containing lysis buffer and reverse transcription mix. Barcoded primers are released via UV exposure and captured poly(A) RNA is transcribed to cDNA before drop breakage. In contrast to Drop-Seq where amplification takes place by PCR of cDNA, inDrop cDNA is linearly amplified via T7 *in vitro* transcription. Next, the *in vitro* amplified and barcoded RNA becomes fragmented and sequencing adapters are ligated before another round of reverse transcription. Resulting cDNA fragments are amplified, sequenced and analyzed (145).

It is worth mentioning that commercial platforms for droplet-based scRNAseq are also available following the same principles. The Nadia instrument (Dolomite Bio) is an automated microfluidic encapsulation device that streamlines Drop-Seq into a user-friendly interface and the possibility of running multiple samples at the same time. ddSEQ™ Single-Cell Isolator (Bio-Rad) is another Drop-Seq-based device that uses polystyrene beads. Finally, the Chromium Single Cell Gene Expression Solution (10X Genomics) also multiplexes up to 8 samples per run and uses hydrogel beads in a microfluidic architecture (using the Chromium Controller, 10X Genomics) more similar to inDrop but with a TSO molecular approach (165). The advantage is that company-based devices provide fully automatic instruments for capturing and molecularly barcoding cells. However, the easier handling comes along with loss of control during the emulsion formation and an increase in costs per cell (Table 3).

Microfluidic-based approaches have two key advantages for the study of tumors and TME. First, they offer abroad range of cell sizes that can be captured which it is ideal for a complex tissue, where it is expected to contain small cell types such as lymphocytes and bigger cells such as macrophages or epithelial cancer cells. The second advantage is their capacity of capturing many cells in a single run and in short time. Thus, these technologies are extremely useful to generate an overall picture of cell types present in a tumor.

### ScRNAseq methods for specific targeted populations

Tumors generally harbor an overrepresentation of epithelial cancer cells, thus in unbiased capture approaches the presence of rare populations is proportional to their abundance, so a large number of cells would need to be sequenced to properly study low-abundant cell populations present in the TME, including some subtypes of IICs like MDSCs. An effective approach to comprehensively study rare immune cells subtypes is enriching for these populations before single-cell capture. Cell surface markers can be targeted using antibodies coupled to fluorophores and subsequent fluorescent activating cell sorting (FACS) to enrich for specific cell populations. For example, the enrichment of MDSCs by sorting for CD45^+^F4/80^−^CD11b^+^Gr1^+^ cells can give a better insight about M-MDSCs and PMN-MDSCs (119).

Droplet-based scRNAseq are suitable for targeted scRNAseq but they require pre-enrichment of the cell population of interest. Staining and sorting of cells might be coupled to cellular activation and/or cellular stress, which might contribute to an altered transcriptome and a decrease in cell viability. Additionally, even after cell sorting, the number of cells recovered could limit the application of subsequent droplet-based techniques. Therefore, these caveats need to be taken into consideration in the experimental design and choice of scRNAseq platform. Drop-Seq involves high flow rates and low cell capture efficiency (144, 173), while inDrop performs at lower flow rates and present much higher cell capture efficiency (145). As an alternative to FACS, an antibody-based cell enrichment approach useful as a preparatory technique before droplet encapsulation is the magnetic cell separation, both targeting the cell population of interest or depleting indifferent and/or dead/dying cells.

Another approach for scRNAseq analysis of targeted cell populations is the use of static-microfluidic devices like the Fluidigm C1 system combined with the CEL-Seq2 (or Smart-seq2 protocol). Here the automatic microfluidics instrument is able to allocate cells into nanoliter wells and subsequently load the CEL-seq2 barcodes (167). Cells are lysed, RNA is reverse transcribed and amplified by IVT before samples are pooled for library preparation.

Finally, there are approaches that directly FACS sort cells in high-density microwell plates. MARS-Seq (Massively parallel single-cell RNA-Seq) is designed to directly correlate FACS sorting profiles with transcriptomics on the same single cell (143). Cells are stained with specific cell surface markers and individual cells are index-sorted into wells of a 384-well plate containing UMI-barcoded primers and lysis buffer. After reverse transcription, cDNA of single cells is pooled, cDNA is *in-vitro* transcribed for amplification and RNA is fragmented and combined with adapters for sequencing of libraries. Through index-sorting MARS-Seq can be used to sort different cell types derived from the same tumor. Paul et al. used MARS-Seq to study the heterogeneity in cells of the myeloid lineage derived from the bone marrow and identified seven groups of progenitors with defined cell fates (174). This overruled the view of hematopoiesis as a progressive loss of differentiation potential along the lineage, however, this study showed that hematopoietic cells undergo lineage commitment at early stages. It proved that MARS-Seq is a powerful tool to study the myeloid lineage and could be used to study this lineage in the TME to reveal alterations in progenitors that are necessary for tumor progression.

### Other high-throughput ScRNAseq methods

Single-cell RNAseq technics are constantly improving. Two recently developed methods combining microfluidics and plate-based approaches are Seq-well (147) and Microwell-Seq (146) enabling to sequence low-input samples on a high-throughput fashion. Seq-well uses arrays of nanoliter size wells that are loaded with barcoded beads and cells by gravity in combination with microfluidics to isolate single cells (147). Microwell-Seq uses agarose-constructed microwells to trap single cells with barcoded beads that are loaded using microfluidics (146). Another recently developed method uses the high-throughput of Drop-Seq in combination with sNuc-Seq (DroNc-seq) that allows sequencing of single nuclei from tissues that cannot be easily dissociated into a single-cell suspension (168). Nuclei are first isolated from a complex tissue and then single nuclei are captured together with a barcode bearing bead using a similar device as in Drop-Seq, however the channel of the microfluidic device is narrower accommodate the smaller size of the nuclei compared to a single cell; but also creates smaller droplets, compensating the lower RNA input from single nuclei compared to single cells. This technique was developed for brain tissues to preserve the integrity of neurons (168) and could have potential to be useful for studying tumor cells and the TME from archived paraffin-fixed samples. Furthermore, this technique has been successfully used for frozen human and mouse brain tissues (168), which could be advantage for patient tumor samples that cannot be processes freshly. Thus, in this context, there is another high-throughput scRNAseq method developed recently, Nanogrid single-nucleus RNAseq, that has been able to obtain scRNAseq from isolated nuclei from archived fresh frozen tumor tissues (148). This automated method (ICELL8 system, Wafergen, Inc.) nanodispenses isolated nuclei into an alloy nanogrid that contains 5,184 nanowells with preprinted oligos containing cell and UMI barcodes and an oligo dT. Then a sophisticated and automated imaging system selects the nanowells that contain one nuclei of the selected size and only those wells will have the reagents deposited to perform downstream cDNA preparation and amplification by SCRB-Seq (175).

All of the above methods are based on the isolation of cells from their physical compartments, this could alter a cell's transcriptome due to the activation and repression of cellular pathways including stress signals. Only recently two methods were developed enabling scRNAseq experiments with fixed cells or extracted nuclei (169, 170). In both of these methods transcripts are labeled in-cell with a combination of different barcodes introduced via split and pool method. Sci-RNA-seq (Single cell Combinatorial Indexing RNA sequencing) developed by Cao et al. in 2017 is able to incorporate a total of three different barcodes after cell fixation and permeabilization with methanol. A first barcode is introduced via poly(T) primer containing also a UMI. After reverse transcription cells are pooled and redistributed, 10–100 cells per well, using FACS where second strand synthesis and tagmentation using Tn5 transposase takes place. Thereby a second index is incorporated via Tn5 adapter. Subsequently cells are lysed and fragments are PCR amplified using a primer binding the barcoded poly(T) primer on the one side and the Tn5 adapter insertion on the other side. Thereby, a third index can be introduced via PCR primer (169). The SPLiT-seq method (Split Pool Ligation-based Transcriptome sequencing) is even able to incorporate up to 4 barcodes enabling parallel labeling of over 1 million cells. Thereby RNA of formaldehyde-fixed cells or nuclei pooled in wells of a 96-well of 384-well plate is reverse transcribed which incorporates the first index via barcoded primer. The second index is introduced using an in-cell ligation reaction and a third barcode containing also an UMI is introduced with a second ligation reaction. Cells are then pooled and split a last time before the introduction of sequencing primers PCR containing a fourth barcode (170). For data analysis, in both methods, sequencing reads that contain the same combination of barcodes are collapsed.

### Analytical computational frameworks for scRNAseq

The boom of single cell transcriptomics has also been encompassed with an equally important and challenging tsunami of development of computational methods and analytical frameworks to extract biological information from the vast amount of information generated by scRNAseq experiments. These computational methods typically aim to assign phenotypic characteristics that can produce biologically meaningful information proposing functional annotation of each of the cells analyzed. In cancer, these methods have been used to propose *in silico* simulations of the tumor composition (116); to resemble the acquisition of hallmarks of cancer progression, such as to model the inflammatory processes that govern the TME (157); to estimate the invasive and metastatic capacity of cancer cells (111, 153, 154); or to study transcriptional rewiring that results in anti-cancer therapy resistance (156).

Despite the wide variety of computational methods to analyse scRNAseq data [reviewed in Zappia et al. (176)] a typical workflow can be depicted (177). At a glance, this computational workflow can be grouped into two stages: (1) Data processing and (2) Data analysis. Data processing involves the manipulation of the sequencing data from a fastq or bcl file to a data matrix of expression values for each gene in each cell, often called “digital expression matrix” (DGE). This stage involves sequence quality control (QC), poly(A) trimming, and alignment to the reference genome for transcript identification, de-duplication and de-multiplexing of barcodes and UMIs, and digital quantification of the expression levels of each gene identified in each cell captured (DGE). The DGE data frame will then be used as an input for the second stage.

The Data analysis stage is by far the most variable in methods as it greatly depends on the nature of the sample and the biological questions to be answered with the dataset, but in general most if not all the computational pipelines will include tools for (1) data normalization and QC; (2) cell clustering and classification using variable genes, marker gene identification, principal component analysis, and dimensional reduction; and (3) cell alignment along functional signatures, pseudotiming modeling, or trajectory analysis (176).

An additional level of complexity on the computational methods consists on overlaying muti-omics data from the same biological entity. This approach enable a much deeper characterization of complex biological tissues and it is in increasing demand, examples of this includes overlaying data from CNV analysis (112, 150, 151, 156, 158), scDNAseq (156), targeted scDNAseq (114, 155), scDNA methylation and scDNAseq (152), or protein information in form cell surface marker definition (143, 174). This approach also allows *in silico* deconvolution of bulk sequencing data not only from RNA (178) but also from genomic data (179).

### Limitations of the 3′ mRNA sequencing-based methods and future solutions

Another important methodological difference is the type of sequencing output that provides each platform. The major limitation of 3′biased methods (Table 3 and Figure 3) is the lack of information of transcript variants. There are several studies demonstrating the importance of alternative splicing in cancer and the immune system and their impact on therapy responsiveness (180–184). One example is the *CD19* gene in leukemia, where an alternative splicing variant impairs patient response to immunotherapy (185). Several immune-related diseases were found to be linked to alternative splicing events [reviewed in Schaub and Glasmacher (184)]. This could also have an impact on the TME in cancer progression. ScRNAseq of full transcripts can not only reveal interesting changes in splice variants in the IICs in cancer but also allows the study of TCR clonality to explain T cell exhaustion in cancer, as previously shown (112). Thus, the most comprehensive scRNAseq to date to give full-length mRNA information is Smart-seq2, thus ideally an adaptation of this into a more high-throughput workflows would be ideal for a complete understanding of tumor biology at the single-cell level. One of these possible adaptations would be to perform long read sequencing. In 3′based methods, the transcript is captured using a polyT oligo, subsequent molecular pipelines process the original full-length transcript and only the cell barcoded and UMI 3′-end of each molecule is sequenced using Illumina short reads sequencing. However, there is a possibility of pairing these 3′-based methods with sequencing methods that allow long reads. Third generation sequencing technologies (TGS) developed by for example Pacific Biosciences (PacBio) and Oxford Nanopore Technology (ONT) allow single molecule long-read sequencing (1–100kb), which allows sequencing of full-length transcripts without fragmentation that is needed for Illumina sequencing (186–190). PacBio has developed single-molecule real-time sequencing (SMRT), in this method hairpin adapters are ligated to both ends of the dsDNA template forming a single-stranded circular DNA molecule. The circular DNA is loaded to a SMRT-cell containing a polymerase and fluorescent-labeled nucleotides, the polymerase binds to the molecule and incorporation of a nucleotide produces a light signal that is recorded (189).

The MinION system from ONT uses nanopores that measure a change in electrical conductivity dependent on the nucleotide when a single DNA molecule passes through the pore (191). This system has been successfully used in combination with the Smart-seq2 protocol to identify transcript variants in mouse B1a cells (192).

## Further use of single cell transcriptomics

Apart from using single-cell RNAseq to analyze transcriptomics to define cell types, its combination with other molecular and bioinformatics technics can increase its power to study immune compartments of the TME. Thus, single-cell transcriptomics can be combined with other omics technics. For example, the combination of single-cell genomics and transcriptomics can help to draw a connection between alterations in the cancer genome and its influences on immune cells. This is possible for example with methods like G&T-Seq, that separate poly-adenylated RNAs from genomic DNA by using biotinylated poly(A) primer prior to sequencing (193). ScTrio-Seq goes even further by combining three omics approaches, genomics, transcriptomics, and epigenomics on the same single cell (152). This has the potential to also study the influence of epigenetic changes on cells of the TME by simultaneous analyzing the genome and transcriptome.

Single-cell RNAseq data could be used for *deconvolution* of bulk RNA-seq data. Deconvolution infers by mathematical modeling the presence and proportion of cells of a specific compartment in a complex tissue based on the expression of reference genes in a certain compartment (178). This strategy can be applied to large databases built from bulk RNAseq data of tumor tissues (such as the TCGA) to analyze the composition of the TME. A deconvolution approach called Epigenomic Deconvolution (EDec) has been developed to *in silico* model the cell composition of complex tissues, in this case breast tumors, based on DNA methylation profiles (179). The algorithm uses reference profiles of a certain tissue to select loci based on different methylation profiles (feature selection). RNAseq data is then dissected into subgroups based on a reference-free *deconvolution* approach based on their molecular profile. These two datasets are then combined to identify cell types by comparing molecular profiles with methylation profiles. With this approach, the proportion of immune cells in breast tumors was inferred and used to predict patient outcome. Single-cell epigenomics data could improve this approach by producing information of methylation pattern of rare cell types to get an even more accurate modeling of cell type compositions.

Another application of scRNAseq data is their usage for reconstructing cell trajectories to follow cell differentiation. To model cell state transitions several bioinformatics algorithms, using unsupervised or supervised (with a little prior information about cell types and marker gene expression) modeling, have been developed to reconstruct cell trajectories (194–196).

A very recent published algorithm is CellRouter, which is very robust in reconstructing trajectories between early cell states and transitory cell states to model cell differentiation (197). CellRouter works in a way that is not dependent on a priori knowledge about cell structure relationships. Single-cell data are first split into subpopulations based on community detection algorithms and then trajectories are determined across subpopulations based on calculated weights, which are weaker between unrelated cell types and stronger within related subpopulations.

ScRNAseq has a great potential to increase our knowledge about immune cancer tolerance to help us find new targets for immunotherapies. However, the main limitation of scRNAseq approaches is that cells are isolated from their environment, making difficult the analysis of connections between cells of different compartments (198, 199). The field of single-cell spatial transcriptomic is of intense research and multiple technologies have been developed in the last few years like seqFISH (200), MERFISH (201), FISSEQ (202), or TIVA (203). SeqFISH and MERFISH use single-molecule FISH (smFISH) techniques, using probes that bind to the same mRNA, but have different fluorophores attached. During several rounds of *in situ* hybridization and stripping off probes RNAs get a unique fluorescent barcode. This allows the simultaneous detection of many transcripts, although theoretically the usage of 4 dyes and 8 rounds of hybridization would cover the whole transcriptome (4^8^ = 65,5336) these methods are based on background information about marker genes that could be provided by scRNAseq data. The FISSEQ (fluorescent *in-situ*) technique uses reverse transcription *in situ* to convert RNA into cross-linked cDNA amplicons followed by a sequencing-by-ligation technique (SOLiD). Finally, TIVA (transcriptome *in vivo* analysis) uses a photoactivatable biotin-labeled TIVA-tags, which upon photoactivation enable mRNA capture from single cells in live tissue. All these methods allow the detection of expressed genes *in vivo* in the context of a specific tissue architecture, and thus inference of the interactions between different cell types. Thus, they are a potential approach to study the connection of epithelial cancer cells and the TME *in vivo* to model the influence of immune cells to cancer cell progression.

## Conclusions

Single cell RNAseq has arisen as the preferred method to understand complex biological systems and thus the number of scRNAseq studies in tumor biology is gaining momentum. Initial studies were focussed on the analysis of cancer cells but the increased capacity of newly developed scRNAseq methods opened the door to the simultaneous study of the composition of tumors, including stromal and immune cells. We predict the number of publications analyzing tumor cell diversity will exponentially increase both in preclinical models and human samples; this will allow, not only a better understanding of the cell types and states present in tumors but also it will set the first roadmap for more focussed studies aiming to increase resolution in specific cell compartments that are still poorly characterized. For example, cancer initiating cells or tumor infiltrated populations of myeloid-derived suppressor cells where canonical studies using the analysis of cell surface markers have not been able to resolve the identity and heterogeneity of these controversial populations. The targeted study of specific cell populations will result in enhanced molecular resolution, useful to determine for example the clonality of reactive and exhausted T-cell species in tumors, a key basic understanding that is greatly needed for the refinement of immunotherapies. Finally, scRNAseq will be the basis to unravel networks of cell-to-cell communication that ultimately could be translated to the clinic, as a measurement of therapeutic response or as targets for novel therapies. We are immersed in very exciting times for tumor biology and the next frontier is the development of devices, tools and methods that will allow us to apply scRNAseq analysis to the routine clinical practice in cancer management.

## Author contributions

FV-M and DG-O organized and directed the manuscript writing. They conceived the outline and rational flow and are the main writing contributors with executive editorial responsibility. KH, AL, and RS wrote specific sections of this review. SO and CO provided critical review and suggestions. All authors proofread the manuscript.

### Conflict of interest statement

The authors declare that the research was conducted in the absence of any commercial or financial relationships that could be construed as a potential conflict of interest.
